# Hydroxychloroquine in systemic lupus erythematosus and psychosis. A case report

**DOI:** 10.1192/j.eurpsy.2024.1558

**Published:** 2024-08-27

**Authors:** M. Martínez Grimal, A. M. Morales Rivero, P. Rivero Rodríguez, E. E. Morales Castellano, N. Molina Pérez

**Affiliations:** Hospital Universitario de Gran Canaria Doctor Negrín, Las Palmas, Spain

## Abstract

**Introduction:**

Hydroxychloroquine, an antimalarial drug, is an important therapeutic tool in the management of rheumatic diseases such as Systemic Lupus Erythematosus (SLE) due to its anti-inflammatory action. SLE is a chronic autoimmune inflammatory disease that affects the connective tissue of multiple organs. Neuropsychiatric disturbances in SLE are common; however, lupus psychosis is rare, occurring in 2 to 11% of patients. The literature has described the emergence of neuropsychiatric symptoms as an adverse effect of hydroxychloroquine use, with some patients experiencing clinical depression, anxiety, suicidal ideation, and psychotic symptoms.

**Objectives:**

The aim of this work is to review the available evidence regarding neuropsychiatric symptoms secondary to the use of hydroxychloroquine.

**Methods:**

The case of a 50-year-old woman diagnosed with SLE, with no other relevant medical history, has been evaluated. She was brought to the emergency department due to paranoid and persecutory ideas, as well as self-referentiality, coinciding with the introduction of hydroxychloroquine in her treatment. She was admitted to the University Hospital of Gran Canaria Doctor Negrín with a diagnostic orientation of a first psychotic episode.

**Results:**

The presence of neuropsychiatric symptoms in patients diagnosed with SLE is so common that they constitute a diagnostic criterion for the disease. On the other hand, the medications used for therapeutic management of this disease can lead to the emergence of new neuropsychiatric symptoms or exacerbate preexisting neuropsychiatric clinical manifestations.

**Conclusions:**

The study of this case highlights the challenges in establishing a differential diagnosis between primary SLE symptoms that require an increase in hydroxychloroquine and those caused by its own treatment. It underscores the need for further studies to explore the risk of psychiatric symptoms associated with the use of hydroxychloroquine, as well as its impact on the course of underlying mental disorders.

**Disclosure of Interest:**

None Declared

